# Perception of vocoded speech in domestic dogs

**DOI:** 10.1007/s10071-024-01869-3

**Published:** 2024-04-16

**Authors:** Amritha Mallikarjun, Emily Shroads, Rochelle S. Newman

**Affiliations:** 1 Penn Vet Working Dog Center, University of Pennsylvania School of Veterinary Medicine, Philadelphia, USA; 2 Department of Hearing and Speech Sciences, University of Maryland, College Park, USA

**Keywords:** Canine speech perception, Degraded speech perception, Vocoded speech

## Abstract

Humans have an impressive ability to comprehend signal-degraded speech; however, the extent to which comprehension of degraded speech relies on human-specific features of speech perception vs. more general cognitive processes is unknown. Since dogs live alongside humans and regularly hear speech, they can be used as a model to differentiate between these possibilities. One often-studied type of degraded speech is noise-vocoded speech (sometimes thought of as cochlear-implant-simulation speech). Noise-vocoded speech is made by dividing the speech signal into frequency bands (channels), identifying the amplitude envelope of each individual band, and then using these envelopes to modulate bands of noise centered over the same frequency regions – the result is a signal with preserved temporal cues, but vastly reduced frequency information. Here, we tested dogs’ recognition of familiar words produced in 16-channel vocoded speech. In the first study, dogs heard their names and unfamiliar dogs’ names (foils) in vocoded speech as well as natural speech. In the second study, dogs heard 16-channel vocoded speech only. Dogs listened longer to their vocoded name than vocoded foils in both experiments, showing that they can comprehend a 16-channel vocoded version of their name without prior exposure to vocoded speech, and without immediate exposure to the natural-speech version of their name. Dogs’ name recognition in the second study was mediated by the number of phonemes in the dogs’ name, suggesting that phonological context plays a role in degraded speech comprehension.

**Supplementary Information:**

The online version contains supplementary material available at 10.1007/s10071-024-01869-3.

## Introduction

Human speech comprehension is robust; even with an extremely degraded signal, humans can still understand the meaning of spoken words and sentences (Remez et al. 1981; Shannon et al. 1995). One form of degradation that has been explored in particular is *noise-vocoded* speech; this signal is of interest because it is thought to replicate the type of signal created by a cochlear implant. The incoming speech signal is divided into a number of distinct, nonoverlapping frequency bands, and the amplitude of each band is used to modulate a band of noise that covers the same frequency region. The combination of these bands results in a signal that can be interpreted as speech, but of an extremely unnatural kind (Shannon et al. 1995). Vocoded speech retains temporal envelope information, but the fine spectral structure cues of speech are lost. Importantly, the degree of spectral degradation depends on the number of bands; as the signal is divided into more bands, more of the spectral resolution in the original signal is preserved, and the easier it is to identify the signal.

Adult humans can generally comprehend vocoded words and sentences even with little or no practice; despite the reduced fine-grain spectral or pitch information, the remaining temporal envelope carries much of the necessary information to understand speech (Davis et al., 2005). Young children and infants can also recognize familiar words in vocoded speech (Newman et al. 2015), demonstrating that they can use temporal envelope cues in isolation as a sufficient cue to speech identity. But whereas adults can interpret a signal made up of as few as 4 frequency bands (Shannon et al. 1995), many toddlers fail to recognize known words at that level, succeeding only when the signal contained at least 8 channels (Newman and Chatterjee 2013).

It is unclear whether nonhuman animals utilize similar cues as humans do to comprehend degraded speech. Comprehension of vocoded speech in nonhuman animals has been explored more often in animals without linguistic experience. Studies have used both rats and chinchillas in different paradigms (Ranasinghe et al. 2012; Shofner 2014; Shofner et al. 2018). With chinchillas, animals were conditioned to a particular speech stimulus during a training period and then subsequently presented with the vocoded version of the stimulus in a testing phase to assess whether they identified the vocoded version as the target stimulus. One study showed that chinchillas failed to identify the vocoded version of a trained syllable at the same rate as humans (Shofner 2014), and a subsequent study demonstrated that chinchillas’ mean recognition rate of a 16-channel vocoded version of a learned word is only about 20%, as compared to human recognition at close to 100% in a similar paradigm (Shofner et al. 2018). This pattern of results suggests that chinchillas are not using the same cues as humans to identify familiar words in degraded speech, or at least that they fail to generalize from the full signal to a degraded one.

The rat study, in contrast, was primarily exploring rats’ ability to discriminate between degraded target words and their minimal pairs (words that differ by only one phoneme); for example, they were asked to discriminate between degraded versions of *dad* and *deed* (Ranasinghe et al. 2012). Here, the participants were specifically trained on vocoded stimuli. This study found that rats did successfully identify trained items in as low as 2-channel noise-vocoded speech and discriminated between the vocoded trained stimulus and vocoded minimal pairs. But while rats can distinguish these 2-channel vocoded stimuli from one another, this does not mean that they relate these signals to natural-speech signals in any way.

Recognition of degraded speech in a nonhuman animal with human language experience has been explored with Kanzi, a language-trained bonobo (Lahiff et al. 2022), and Panzee, a language-trained chimpanzee (Heimbauer et al. 2011, 2021). In similar paradigms, both recognized noise-vocoded tokens of familiar words and correctly mapped them to the corresponding target image or pictogram, presented in sets of three (Lahiff et al. 2022) or four (Heimbauer et al. 2021). In Heimbauer et al. (2011), Panzee achieved above-chance performance (55% accuracy, with chance at 25%) with spontaneous recognition of 7-channel noise-vocoded stimuli during her first exposure to the stimuli; this provides some evidence that language experience contributes to the ability to perceive degraded speech. In the follow-up study, she subsequently was above chance for more degraded noise-vocoded speech between 3 and 5 channels, only dropping to chance with 2-channel vocoded speech (Heimbauer et al. 2021). During test trials, Kanzi’s recognition of noise-vocoded speech was also above chance (62.5% accuracy, with chance at 33.3%); however, unlike Panzee, Kanzi was provided with training in which he saw natural stimuli alongside noise-vocoded stimuli and was provided with feedback on his selections (rewarded for selecting the correct pictogram response for the associated stimuli). Training phases here served multiple functions; Kanzi was taught that he should attend to the noise-vocoded speech, and, as stated in Lahiff et al., “in line with previous work (Heimbauer et al. 2011), before testing we exposed Kanzi to training programs to help him learn that the test stimuli can be processed in the same way as unmanipulated human speech”. Prior experience with vocoded speech has been shown to improve humans’ performance on vocoded speech perception (Davis et al., 2005); as such, prior experience is one possible reason for Kanzi’s success at this task.

Given the potential impact of prior language experience and familiarity with speech, it is worth noting that Panzee heard degraded speech derived from the speech of someone very familiar to her. Familiarity also can impact speech recognition performance in humans, where familiarity with the person producing target speech facilitates understanding; however, it remains unclear the extent to which this familiarity effect remains when listening to a degraded signal, particularly given that many of the cues to talker identity are reduced in the process of vocoding.

The test sessions for both Kanzi and Panzee contained both vocoded speech trials as well as natural speech trials for performance comparison; the inclusion of interspersed natural speech trials could increase performance, as listening to the natural speech version of a word could facilitate the comprehension of the same word in noise-vocoded form (Giraud et al. 2004). However, since the word order was randomized in all studies, this facilitation likely only occurred for a subset of their total word set. The inclusion of natural speech in general could also facilitate speech comprehension by increasing the listeners’ attention to the stimuli, which improves degraded speech comprehension (Huyck and Johnsrude 2012). In sum, Kanzi and Panzee’s task success could be partially attributed to the inclusion of interspersed natural speech stimuli, as well as their prior language experience, their close evolutionary relation to humans as compared to chinchillas and rats, or a combination of these factors; further, Kanzi may have experienced increased performance due to his training experience with vocoded speech prior to test.

Dogs are not as closely related to humans as bonobos or chimpanzees, but have unique exposure to human speech from living alongside humans. Pet dogs overhear speech in their everyday life, and people often direct speech to dogs (Ben-Aderet et al. 2017; Benjamin and Slocombe 2018). Dogs can quickly learn a vocabulary of commands and learn related words from exposure. As a result, dogs serve as a useful model to test questions addressing the aspects of speech perception that are human-specific, and the aspects that are derived from more general cognitive processes. Examining dogs’ recognition of vocoded speech can address whether non-human animals can use the reduced information from the degraded speech to identify familiar words and whether they naturally rely on similar acoustic cues for recognition as do humans.

## Experiment 1: Name recognition with both vocoded and natural speech

In this experiment, dogs were presented with their name and another dog’s name in both natural speech and the vocoded versions of this natural speech. Prior studies have demonstrated that dogs will listen longer to their name than to another dog’s name, even when spoken by a novel talker (Mallikarjun et al. 2019). By examining whether they continue to do so with a noise-vocoded signal, we examine whether their recognition of their own name generalizes to a very different (degraded) acoustic signal. If so, it would suggest that at least some of the acoustic cues dogs use to recognize their name remain present in a vocoded signal; this, in turn, would suggest that dogs are perceiving speech using similar cues as are human adults.

### Participants

Twenty-eight pet dogs (13 male) participated in this study. To participate, dogs were required to have no known prior exposure to any form of degraded speech (e.g., noise-vocoded speech, sine-vocoded speech, low-pass filtered speech). Dogs were additionally required to have had their name for at least ten months prior to participating, not be on any psychiatric medication, and have no owner-reported signs of hearing loss. Three dogs were dropped from the study due to owner interference during testing (1) or noise outside the experiment room distracting the dog (2), for a total of 25 participating dogs (11 male). Participating dogs were, on average, 4.6 years old (*SD* = 3 years), and had heard their name for 2.8 years (*SD* = 2.5 years). Five dogs had a one-syllable name (e.g., Prince) and 20 dogs had a two-syllable name (e.g., Bruno). Further dog demographic information is available in Supplementary Table 1.

**Fig. 1:**
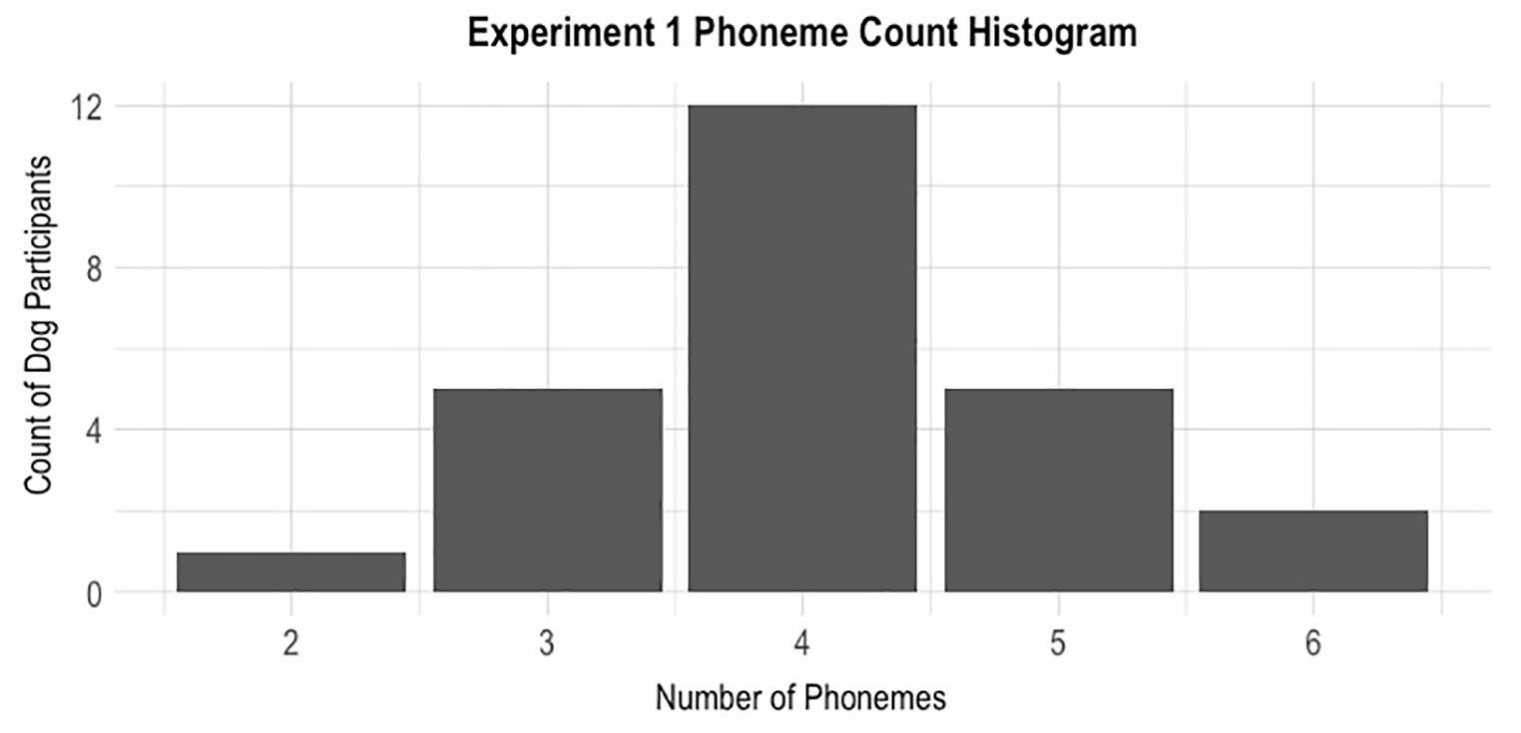
A histogram showing the number of dogs in Experiment 1 by the number of phonemes in their names

In Fig. 1, a distribution of the number of phonemes in each dogs’ name can be seen. Prior research has shown that the more context available in degraded speech, the easier it is to comprehend (Hervais-Adelman et al. 2008; Sheldon et al. 2008). Longer words are less likely to share a lot of sounds with other words, thus are more distinct and easier to recognize (Pitt and Samuel 2006). Together, this suggests that an increase in the total number of phonemes per name could allow for easier comprehension if the speech is degraded.

A sample size calculation was performed in GLIMMPSE (Kreidler et al. 2013) using data from a similarly structured study (Mallikarjun et al. 2019) and 21–33 dogs were needed for a power of 0.8 at an alpha level of 0.05 to see a main effect of name preference (the range was calculated using different means and variability scaling values).

### Test materials

#### Original speech recordings

Stimuli consisted of 15 repetitions of one of two names: the dog’s own name, or that of another dog not from their household (“foil” names or “foils”). These foil names were selected from other dogs that participated in either this study or another related study in our lab. Prior to the study appointment, the owner was asked to provide the most common name or nickname used for the dog. A female native English speaker recorded a stream in which the dog’s name was repeated in lively, dog-directed speech; this formed the Name stream. Each name was matched with a foil name (either a previously recorded dog name, or a future study participant’s name). The foil was selected to match in stress pattern but to be otherwise phonetically dissimilar from the target name.

Fifteen name tokens were selected from each of the recordings of the target name and foil to generate the name and foil streams. The name and foil streams were matched as closely as possible for pitch, duration, intonation contour, emotionality, and voice quality. Pauses between tokens of dog names were adjusted so each stream was a total of 22 s long. Each file had an initial silence period of 0.5 s.

The files were then adjusted to the same amplitude. Since the streams contained silence between each name or foil token, and the overall amount of silence in the target and foil streams was not necessarily identical, a measure of intensity across the file would have been potentially misleading. As such, to eliminate any influence of the silent periods on amplitude measurements, a copy was created of each name stream in which all the pauses between name tokens were removed. Average RMS amplitude was measured across this speech-only file, and necessary amplitude changes were calculated and applied to the original stream containing pauses. In this way, the name and foil streams could be amplified such that the speech, rather than the entire stream, matched in average amplitude. These then served as the natural-speech stimuli.

#### Vocoded stimuli

Noise vocoding was performed using methods akin to published standards (Shannon et al. 1995). The natural-speech files were first band-passed to only include spectral information across the frequency range from 200 to 8000 Hz. The signal was then split into 16 equally-spaced frequency bands using bandpass filtering (Butterworth filters, 24 dB/oct roll-off) and the envelope of each band was extracted using half-wave rectification and low-pass filtering. The envelope derived from each band was then used to modulate a white noise signal with the same bandwidth as the original signal band. This removed the fine spectro-temporal structure within each frequency band. The resulting modulated noises were combined at equal amplitude ratios to create the final 16-band noise-vocoded stimuli. We selected 16-band stimuli for several reasons: first, it is within the range that most studies have selected as a starting point for testing (Newman and Chatterjee 2013; Ranasinghe et al. 2013). Second, while prior studies have shown that human toddlers can recognize speech with as few as 8 channels, and a bonobo and a chimpanzee with 7 channels, chinchillas generally failed to recognize words even with 16 channels. Thus 16 channels seemed like a reasonable test case for dogs’ perception of degraded speech. Example stimuli (ESM_1.wav and ESM_2.wav) are included in the supplementary material.

### Apparatus

**Fig. 2:**
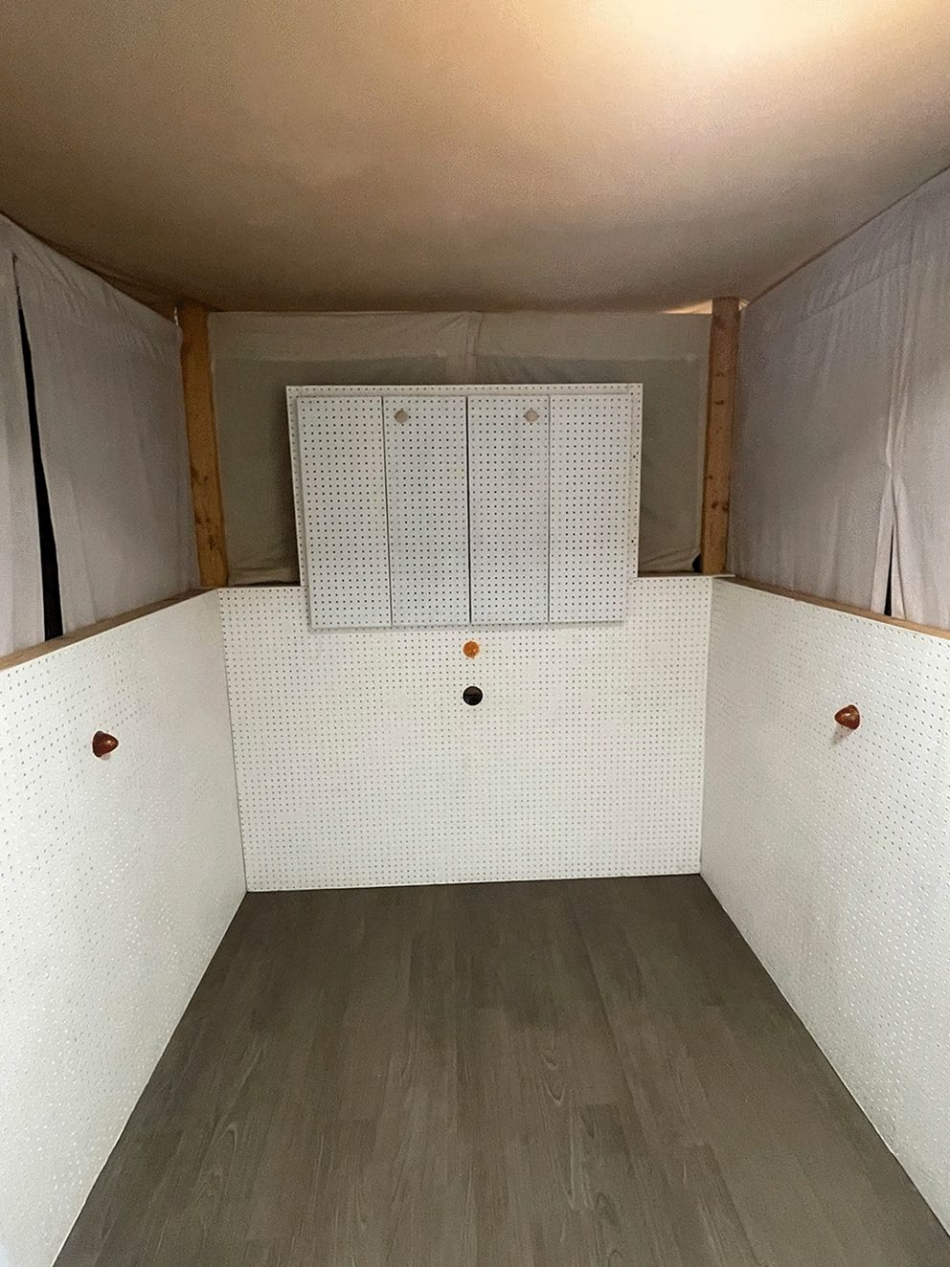
An image of the testing booth

The study took place in a 6-foot by 6-foot three-sided test booth made of pegboard (see Fig. 2). In the front of the booth, there was a hole for a GoPro camera. Above the camera, a light was mounted in the center of the panel. The GoPro recorded each session and allowed the coder to see the dog’s behavior inside the booth. The side walls each had a light mounted in the center and a speaker directly behind the light to play the stimuli for the dog. A curtain hung from the ceiling to the top of the booth to ensure that the dog could not see over the booth. A Windows computer was used behind the front wall of the booth for running the study and coding the dog’s behavior. The experimenter used BITTSy, an experimental program designed for Headturn Preference and other infant looking-based studies, to run the experiment and code the trials (Newman et al. 2021).

### Procedure

Each dog either sat on their owner’s lap or directly in front of the owner, depending on the dog’s size and the owner’s opinion as to what would be most comfortable for them and the dog. The dog could choose to sit facing toward the camera (facing the front of the booth) or toward the owner (facing the back of the booth). In either case, the dog’s attention was maintained (as much as possible) at a point equidistant from the two sides of the booth where the loudspeakers were located. As a result, the dog’s natural inclination, upon hearing a sound through a loudspeaker, was to turn its head 90° to face that sound source.

Owners wore Peltor headphones and listened to classical music at a volume where they reported they could no longer hear outside speech and thus would not be able to cue their dog. They were also instructed to ignore their dog for the duration of the study. If the owners touched their dog or made verbal/physical gestures to attract their dogs’ attention during the study, the dog was dropped from the study.

Dogs took part in a two-trial practice phase prior to the test phase to familiarize them with the situation and task. This practice phase used music as the auditory stimuli rather than anything related to the study question. Two experimenters ran the study: one to code the dog’s looks (the coder), and the other to produce auditory attention getters. At the start of the practice trials, the light on the front of the booth turned on, and one experimenter made a sound to attract the dog’s attention towards the front of the booth. Once the dog attended to the front, a light on either the left or right side of the booth turned on. The experimenter then made a sound on that side. Once the dog looked to that side, a trial began, and one of two instrumental music sound files played from the speaker on that side; the light provided a visual indication of the “source” of the sound (Kemler Nelson et al. 1995).

The coder used a keyboard to code the dog’s looks towards and away from that side. A dog was considered to be looking towards a particular side of the booth if their head turned at least 45 degrees from the center position towards the appropriate side of the booth. The stimulus played for a full twenty-two seconds, or until the dog looked away for two consecutive seconds - whichever occurred first. A dog was considered to be looking away from the stimulus if they turned at least 45 degrees away from the sound source. Any time the dog spent looking away was subtracted from the dog’s overall looking time. The coder wore Peltor headphones playing masking music so she would not be able to hear the auditory stimuli and have that influence her coding. This familiarization phase demonstrated the source of the audio to the dog and also the idea that looking towards the speaker generates additional sound stimulus, and looking away makes the stimulus stop.

The test phase consisted of 16 trials, divided into 4 blocks. Blocks consisted of one trial for each of the four streams. The presentation order of the streams was randomized within each block. The trials were run in the same manner as described above for the practice trials, except that the auditory stimulus was randomized between four options: Natural-speech Name, Natural-speech Foil, Vocoded Name, Vocoded Foil.

### Coding and reliability

In the Headturn Preference Procedure (HPP), trials begin and end based on looking behavior. As a result, the study had to be coded in real time. All coders were first trained to live-code infant HPP studies, where the original coding instructions state that coders must press a button when the participant looks at least 30 degrees towards the stimulus location, which was marked by a flashing light (Kemler Nelson et al. 1995).

As discussed in Mallikarjun et al. (2022), the dog coding process comes with some different challenges than infant coding; however, with practice, coders can easily determine when a dog is paying attention and looking at the proper location, and when the dog becomes bored and turns away. Unlike infants, dogs do not always like to look directly at the light on the side wall and tend instead to train their gaze anywhere on the wall where the speaker is located. As a result, they often will not turn 60–90 degrees to face the side wall speaker, but instead will look 45 degrees to the front or back corners of the booth. The dog was only considered to be attending to the stimuli if the dog’s eyes were facing the wall with the speaker playing the target stimuli (approximately a 45 degree turn from the center position).

While dog attention is partially a judgment call on the part of the coder, it is consistent across coders. Results from prior HPP dog studies showed that inter-rater reliability is high (Mallikarjun et al. 2019, 2020, 2022): using a Pearson’s correlation analysis, the three previous studies had correlation coefficients of 0.93, 0.91, and 0.88 between the first coder and second coder.

All coders in the current study had demonstrated coding reliability before coding any actual participants. Nonetheless, to ensure reliability a second individual re-coded 64 trials from four dogs. An inter-rater analysis comparing looking time from two coders was run in R using the IRR package (Gamer et al. 2019). The IRR was obtained using a single-rating, consistency, two-way mixed effects model. The model shows that the intraclass coefficient was 0.96; this is considered excellent reliability (Koo and Li 2016).

### Model

All statistical analyses were carried out in R, version 4.2.0.

A linear mixed-effects model was used to examine the effect of Name versus Foil, Natural Speech versus Vocoded Speech, and Phoneme Number (the number of phonemes in each dog’s name) on Looking Time. We included phoneme number as a factor because of our use of degraded speech; vocoding tends to preserve some acoustic features more than others (in particular, it preserves temporal cues, such as those found in voicing and manner, more than spectral cues, such as those found in place of articulation; see McGettigan et al. 2014). As a result, some phonemes are more likely to be discriminable than are others. With the presence of more phonemes, there is a greater likelihood of some phonemes being more easily perceived/discriminated. In this sense, length in phonemes can be thought of as a proxy measure for the amount of acoustic information available. The model was done using the lmer() function in R. The fully specified model included Block, Trial, Age, and Length of Time with Name as random intercepts, as well as Block by Dog as a random slope. Aikaike’s information criterion (AIC) was calculated for the fully specified model and reduced models to determine the most parsimonious model. Models that converged were compared. The model with the lowest AIC value was chosen as the final model. The model selected contained Name versus Foil, Natural Speech versus Vocoded Speech, and Phoneme Number as fixed effects, as well as Dog, Block, and Trial as random effects. Addition of other random intercepts and slopes did not improve model fit.

Model assumptions were checked using the DHARMa package (Hartig 2016). The residuals were found to be non-normally distributed and different groups were found to have non-equal variances, so the outcome variable, Looking Time, was transformed using the log function. Subsequently, the model was found to have normally distributed residuals, equal variances across groups, and the outcome variable was normally distributed.

### Results

Table 1 shows the mean looking times across trial blocks. Dogs’ mean looking times to their name were higher than the looking times to the foil names, across all blocks.

**Table 1:** Average Time Across Blocks (sec)

	Natural Speech Name (sec)	Natural Speech Foil (sec)	Vocoded Speech Name (sec)	Vocoded Speech Foil (sec)	Name Total (sec)	Foil Total (sec)
Block 1	12.79	10.83	11.08	10.91	11.94	10.87
Block 2	8.80	5.61	7.70	5.08	8.25	5.34
Block 3	8.64	5.45	7.26	6.24	7.95	5.84
Block 4	8.11	8.23	5.82	4.38	6.97	6.31
Average Across Blocks	9.45	7.30	7.91	6.62	8.68	6.96

#### Model analysis

There was a significant main effect of Natural Speech versus Vocoded Speech, *F*(279) = 3.93, *p* = 0.048. The effect size, calculated as partial eta squared (η²), was 0.01, indicating a small effect. That is, dogs preferred to listen to real speech (a familiar signal) compared to the highly-unnatural vocoded speech. There was a significant main effect of Name versus Foil, *F*(279) = 11.43, *p* = 0.0008, with η² = 0.04, suggesting a small effect. Dogs listened longer to their own name than another dog’s name, replicating prior studies suggesting that dogs both know their own name and can recognize it when spoken by novel voices (Mallikarjun et al. 2019, 2020). There was also a significant main effect of Phoneme Number, *F*(90) = 10.91, *p* = 0.001, with a medium effect size, η² = 0.11, such that overall, dogs with more phonemes in their name listened to the auditory stimuli for less time. It is unclear what this result implies, as it is a difference across different dogs, but since the other variables are all within-subject, the dogs’ differing overall looking times does not impact their within-subject preferences.

Surprisingly, there was no significance in any interaction (Name x Vocode: *F*(279) = 0.013, *p* = 0.909; Name x Phoneme: *F*(279) = 0.601, *p* = 0.439; Phoneme x Vocode: *F*(279) = 1.789, *p* = 0.182; Phoneme x Name x Vocode: *F*(279) = 0.084, *p* = 0.772).

**Fig. 3:**
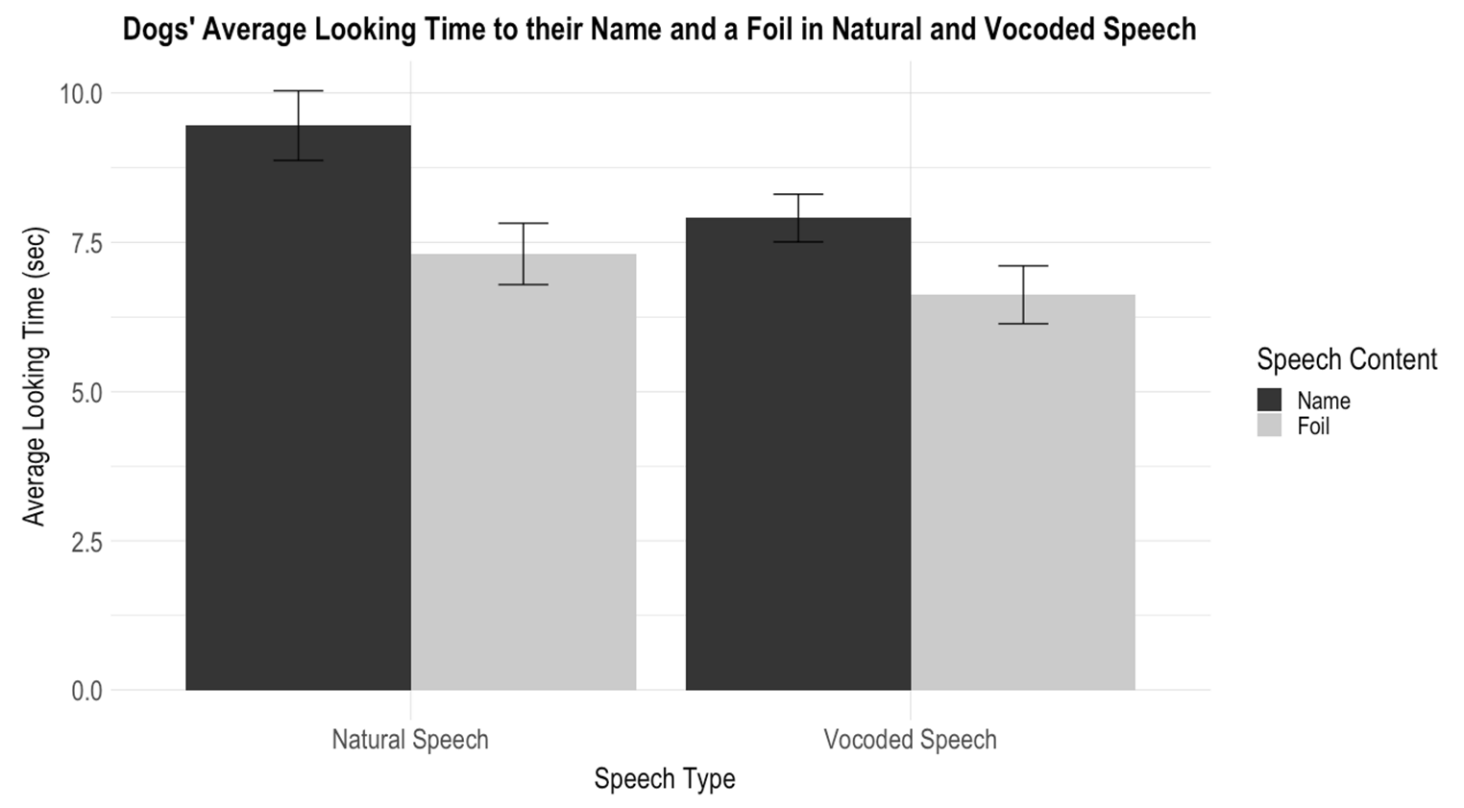
A graph showing dogs’ mean looking time to their name and a foil name in natural speech and in 16-channel noise-vocoded speech. The error bars show standard error. Dogs listened significantly longer to their name than the foil name, regardless of the speech type. Dogs also listened significantly longer to the natural speech than the vocoded speech, regardless of speech content

This demonstrates that regardless of Vocode status and the number of sounds in the dogs’ names, dogs listened longer to their name (*M* = 8.87 s, *SD* = 9.39) over the other dog’s name (*M* = 7.13 s, *SD* = 8.41) for the duration of the study. In other words, dogs continued to listen longer to their name than to a foil name even with a degraded signal. Figure 3 shows dogs’ looking times to their name and a foil in both natural and vocoded speech.

To further support this interpretation, a subgroup analysis was performed examining only the Vocoded trials using the same linear mixed-effects model as above with a post-hoc Tukey correction using the emmeans package. This analysis shows that dogs listened significantly longer to their name than another dog’s name in the vocoded trials, *t*(279) = -2.47, *p* = 0.014, as well as the full speech trials, *t*(279) = -2.31, *p* = 0.022. Thus, the lack of an interaction in the primary analysis was not simply the result of a lack of power – rather, dogs truly recognized the difference between a degraded form of their name and a degraded signal representing the name of another dog.

What is less clear is *how* dogs succeeded. One possibility is that they recognized the vocoded version of their name as being familiar in content, or that they generalized their familiarity with the sounds of their name to even this highly degraded signal.

But it is also possible that dogs were *learning* to interpret this degraded signal during the course of the experiment. Because dogs heard both natural speech and vocoded speech versions of the same recordings in this study, they might have recognized the acoustic similarities across trials. Studies with adult humans suggest that intelligibility of distorted speech (e.g., sine-wave speech or vocoded speech) dramatically increases when people hear the natural version of the sentence prior to hearing the distorted version (Davis et al., 2005). Perhaps the dogs are learning to pattern-match the vocoded versions through a direct comparison across trials. Pattern-matching from natural speech to the vocoded version of that natural speech could be easier than detecting and understanding degraded speech via the underlying acoustic structure itself.

One way to examine this would be to only look at the subset of vocoded trials that occurred prior to hearing a natural-speech trial. Because of the randomized order of trials within a block, roughly half the dogs heard their name in the vocoded condition before hearing it in the natural-speech condition. However, since this would require looking only at the first block of trials (by definition, vocoded trials in blocks 2–4 occurred after hearing the natural-speech version from block 1), and would require looking at only (approximately) half the dogs, there is not enough power for an analysis.

**Fig. 4:**
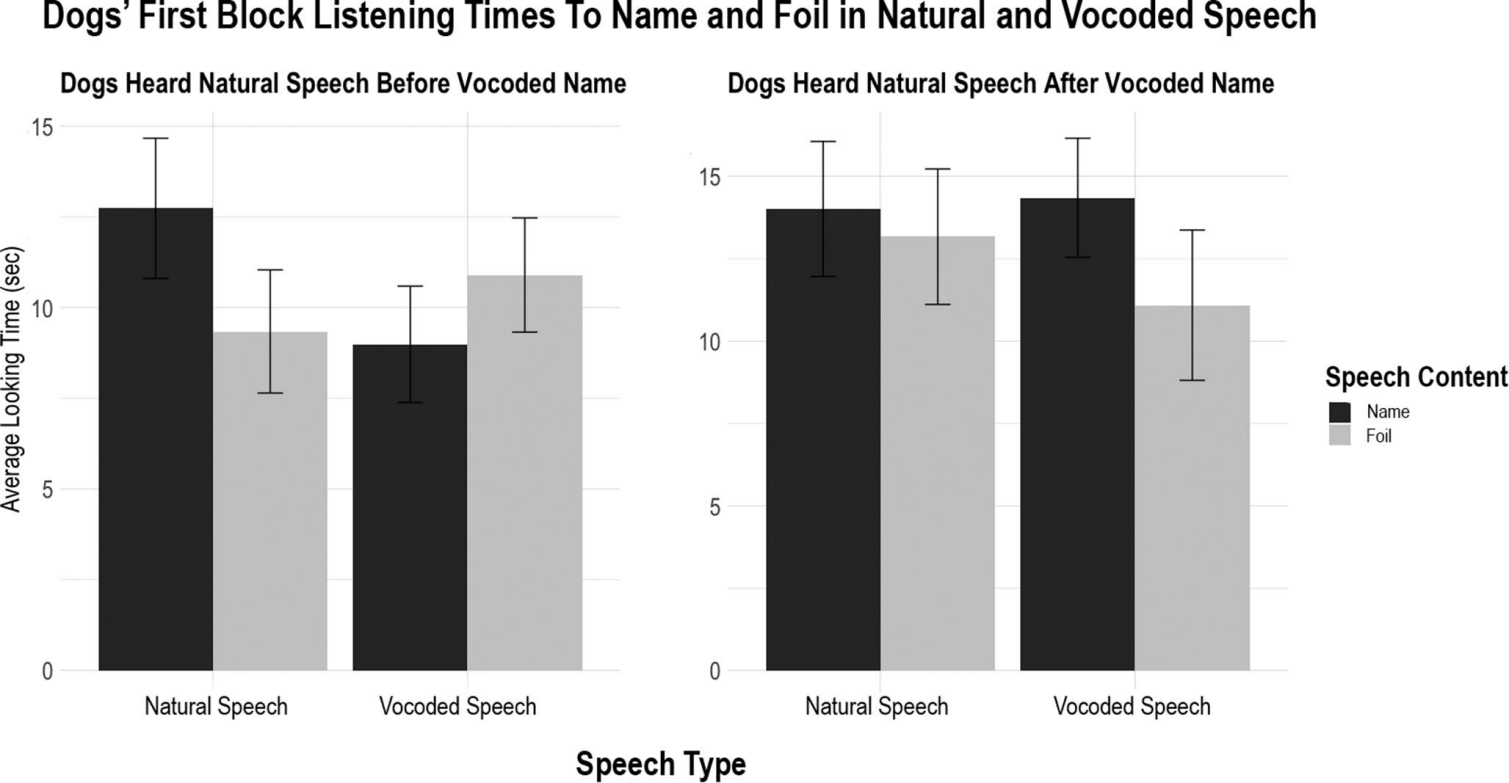
Both panels show dogs’ first-block listening times to their name and a foil in natural speech and vocoded speech. The panel on the left shows the set of dogs that heard their natural speech name prior to the vocoded name (and could thus use potentially rely on pattern-matching across trials). The graph on the right shows the set of dogs that heard the vocoded version of their name before the natural speech version. This error bars display standard error. The dogs’ first block performance was highly variable, making it difficult to assess whether dogs who heard natural speech before vocoded speech used the natural speech information to comprehend the vocoded speech

The graph below shows dogs’ looking times during the first block of trials to names and foils in natural speech and vocoded speech, and splits the dogs into the group that heard the vocoded condition first (right), and those that heard the natural speech condition first (left; see Fig. 4). There is a great deal of variability, making it unclear whether dogs relied on the natural speech to comprehend the vocoded speech.

Thus, this first experiment demonstrates that dogs can recognize their name in vocoded speech, but not whether they can do so without prior familiarization with natural speech tokens as a comparison. To further explore whether dogs can differentiate their name from other dog’s names in vocoded speech alone, we conducted a second experiment in which dogs never heard any clear speech tokens.

## Experiment 2: Vocode-only name preference

In this experiment, dogs were again presented with 4 trials per block for four blocks. But rather than hearing both vocoded speech and natural speech, the dogs were presented only with vocoded speech. The trials consisted of either their name in vocoded speech, or one of three vocoded foil names. If dogs show a preference for their vocoded name in comparison to the vocoded foil names, it suggests that dogs can extend their prior speech representation of their name to a highly degraded signal.

### Participants

**Fig. 5:**
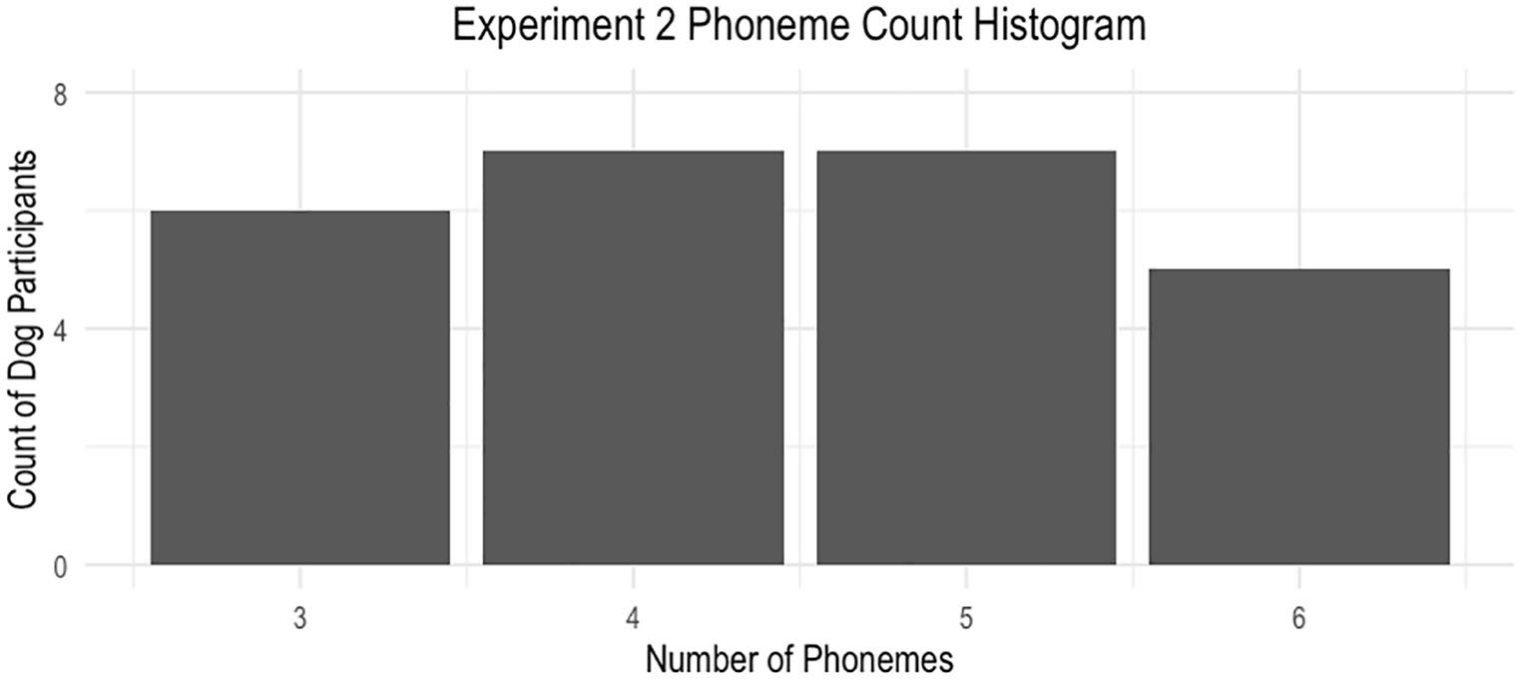
A histogram showing the number of dogs in Experiment 2 by the number of phonemes in their names

Thirty-one pet dogs (15 male) took part in this study. Data from six dogs were dropped due to equipment malfunction (2), unwillingness to participate in the study (2), or not fitting age criteria (2), for a total of 25 participating dogs (15 M). On average, participating dogs were 3.59 years old (*SD* = 2.74 years) and had heard their name for an average of 3.10 years (*SD* = 2.28 years). Three dogs had a one-syllable name (e.g., Tag), and 22 dogs had a two-syllable name (e.g., Toby). The same participation requirements as Experiment 1 applied. Figure 5 shows a histogram of dogs by the number of phonemes in their name. As previously mentioned, more phonemes in a name could allow for easier comprehension if the speech is degraded.

A sample size calculation was performed in GLIMMPSE (Kreidler et al. 2013) using data from a similarly structured study (Mallikarjun et al., 2021) and 20–30 dogs were needed for a power of 0.8 at an alpha level of 0.05 to see a main effect of name preference over foil names (the range was calculated using different mean scaling values and variability scaling values).

### Test materials

The materials were generated in the same way as the Vocode streams from Experiment 1. In this experiment, rather than presenting the dogs with natural speech and vocoded speech, they were presented with vocoded speech only. Dogs heard their vocoded name and three vocoded different foils. The foils were all selected to match in stress pattern and number of syllables, and to be as phonetically dissimilar as possible from the target name. For example, participant dog Toby would hear his name (Toby) as well as three dissimilar foils (Onyx, Jasper, and Baldwin). The foil recordings were selected from prior recordings made for either this study or similar name studies occurring in our lab.

### Apparatus

Same as Experiment 1.

### Procedure

Same as Experiment 1, with Vocoded Name and three different Vocoded Foils as the four trial types.

### Coding and reliability

The coding was done in the same manner as Experiment 1.

To ensure reliability, 56 trials from 4 dogs in this study were coded by a second coder. As in Experiment 1, looking time was compared between coders. The inter-rater analysis was run based on a single-rating, consistency, two-way mixed effects model. The model shows that the intraclass coefficient was 0.90; this is considered good reliability (Koo and Li 2016).

### Model

All statistical analyses were carried out in R, version 4.2.0.

A linear mixed-effects model was used to examine the effect of Phoneme Number and Name versus Foil on Looking Time. The model was done using the lmer() function in R. The fully specified model contained Age, Length of Time with Name, Block, Trial, and Dog as random intercepts, as well as Block by Dog as a random slope. The model with the lowest AIC value was chosen as the final model.

The model selected contained Phoneme Number and Name versus Foil as fixed effects, and Block, Trial, and Dog as random intercepts. Addition of other random intercepts and slopes did not improve model fit.

Model assumptions were checked using the DHARMa package (Hartig 2016). The residuals were found to be non-normally distributed and different groups were found to have non-equal variances, so the outcome variable, Looking Time, was transformed using the log function. Subsequently, the model was found to have normally distributed residuals, equal variances across groups, and the outcome variable was normally distributed.

### Results

Table 2 shows the mean looking times across trial blocks. Here, dogs looked longer at the vocoded name over the foil in three out of the four blocks.

**Table 2:** Mean time across blocks by trial type for Experiment 2

	Vocoded Speech Name (sec)	Vocoded Speech Foils (Mean) (sec)
Block 1	8.23	8.27
Block 2	6.94	4.78
Block 3	6.44	4.85
Block 4	5.96	4.80
Average across Blocks	6.89	5.68

There was a main effect of Name versus Foil such that dogs listened significantly longer to their name (*M* = 6.89 s, *SD* = 4.87 s) than the foil names (*M* = 5.68 s, *SD* = 5.69 s), *F*(381) = 6.40, *p* = 0.012; however, the effect size was close to 0, suggesting a very small effect (η² = 0.005). There was no significant main effect of Phoneme Number, *F*(381) = 0.80, *p* = 0.37.

There was, however, a significant interaction between Name versus Foil and Phoneme Number, *F*(381) = 8.69, *p* = 0.003, with a small effect size, η² = 0.02. As the number of phonemes in the dogs’ name increased, dogs listened to their name more in comparison to the foils.

**Fig. 6:**
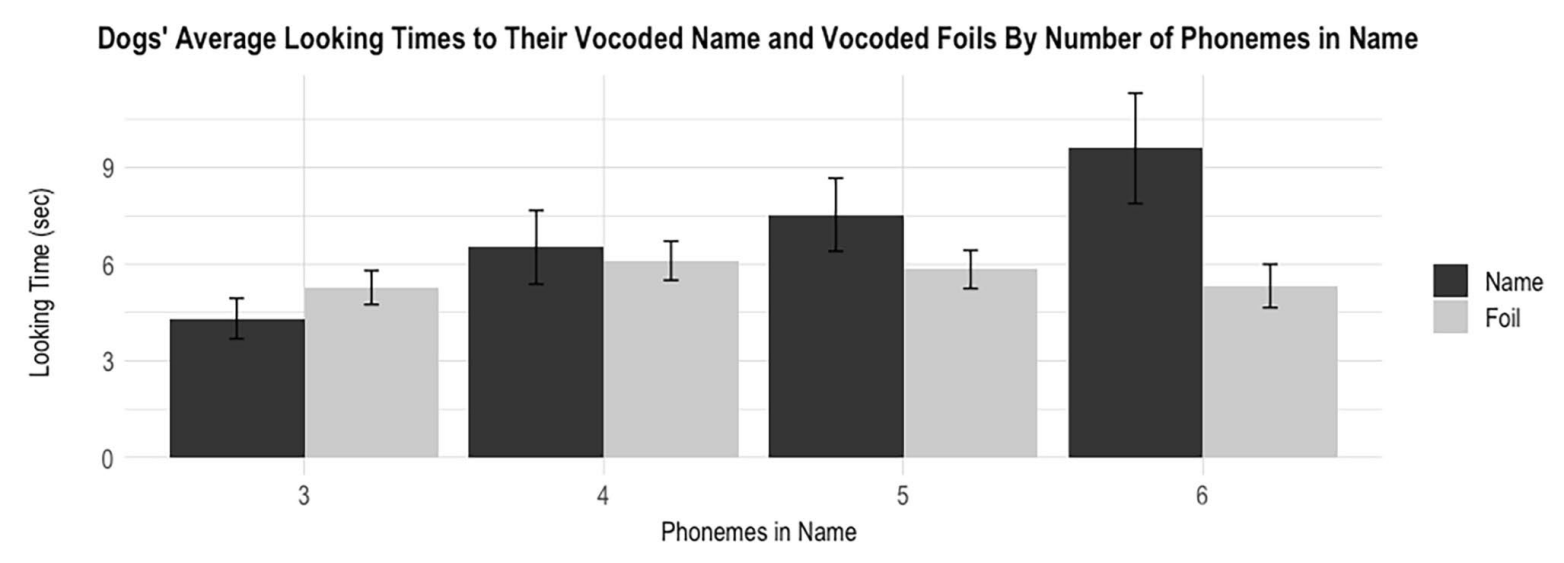
Dogs’ mean looking time to their vocoded name and vocoded foils by number of phonemes in the dog’s name

Together, these results demonstrate that perception of vocoded speech without the full speech analogues is difficult, but the more phonemes present in the speech, the easier it is for dogs to recognize their name and differentiate it from the foils. Figure 6, below, shows the difference in looking time between the averaged name trials and averaged foil trials for each dog with each name length.

### Discussion

This experiment examined dogs’ ability to comprehend vocoded speech without the natural-speech versions of the vocoded speech. Dogs listened longer to their own vocoded name in comparison to vocoded foils; this demonstrates that they recognized their name without the use of pattern-matching to the natural speech versions of their names and foils. Since the name and foils were matched for syllable length, stress, and prosody, and were produced by the same speaker, dogs had to use sound information from the vocoded speech to recognize it. Either dogs can utilize the limited information provided from the vocoded speech to recognize their name, or their high familiarity with the sounds in their name allowed them to recognize the limited content of the vocoded speech.

## Overall discussion

This set of experiments had three primary findings. First, dogs can recognize their name in vocoded speech when they are also presented with a natural speech version of the vocoded speech. Second, dogs can recognize their name in vocoded speech *without* the presence of the natural speech version, suggesting that they can use features of the vocoded speech to recognize familiar words. Third, across both studies, dogs’ high level of performance was achievable with no prior training on or experience with this type of signal; however, given that the number of phonemes in the dogs’ name affects their ability to differentiate their name from foils, it appears that context can play a role in dogs’ understanding of familiar vocoded speech. Each of these findings are discussed below, as well as future potential research directions.

The first experiment found that dogs listened significantly longer to their own 16-channel vocoded name than the foil vocoded name when presented with both stimuli in natural speech and vocoded speech. This is similar to the stimulus sets used with Kanzi, a bonobo, and Panzee, a chimpanzee, where interleaved natural speech and vocoded speech were used in forced-choice paradigms (Heimbauer et al. 2011, 2021; Lahiff et al. 2022). As was the case in prior degraded-speech processing studies using nonhuman animals, natural speech was included in this study because the intelligibility of distorted speech (e.g., sine-wave speech or vocoded speech) dramatically increases when people hear the natural version of the sentence prior to hearing the distorted version (Davis et al., 2005). As such, we chose to include natural speech to facilitate dogs’ performance. Dogs are succeeding in a paradigm where chinchillas failed; even when trained on the natural speech target words, chinchillas failed to identify these words when vocoded with 16 channels (Shofner et al. 2018).

While it is clear from the first experiment that dogs listened longer to their vocoded name than to the vocoded foil name when the natural speech version was also presented, it was not possible with that study design to separate the possibilities that (1) dogs used the information present in vocoded speech to comprehend their name, or (2) dogs matched the acoustic patterns from their natural speech name to the vocoded version without necessarily being able to comprehend the speech directly from the vocoded version. To distinguish between these two possibilities, a second experiment was conducted in which a new set of dogs heard only their vocoded name and three different vocoded foils. The second experiment found that dogs differentiated their 16-channel vocoded name from other dogs’ vocoded names without natural-speech versions of the stimuli. This suggests that dogs can use the cues present in the vocoded speech alone to recognize familiar words.

Importantly, dogs in the second experiment listened longer to their vocoded name without any prior training or exposure to degraded speech of any type. This suggests that the existing information in the 16-channel noise-vocoded speech suffices for dogs’ speech comprehension and that they can quickly recognize and utilize this information. This is in line with other studies from our lab demonstrating that dogs listen longer to their names than foil names in another version of degraded speech: speech in noise (Mallikarjun et al. 2019). In all but one of the prior vocoded speech comprehension studies done with non-human animals, the target species were exposed to vocoded speech in a training/orientation period prior to testing their comprehension (Heimbauer et al. 2021; Lahiff et al. 2022; Shofner 2014; Shofner et al. 2018). Panzee the chimpanzee was able to spontaneously recognize vocoded speech in a paradigm that also included trials with natural speech, as was tested in Experiment 1 with the dogs. The impact of the presence of natural speech is not clear, but it could potentially facilitate attention and in the cases where a natural speech trial for a particular word precedes the vocoded speech trial, could facilitate perceptual performance. In our Experiment 2, there was no natural speech present, and it is unclear whether other species would also spontaneously recognize degraded speech as do dogs, or whether they require the presence of natural speech or a period of training with the degraded speech.

As the number of phonemes in the dogs’ names increased, the difference in dogs’ looking times between the name and the foil names increased in Experiment 2 (Vocode Only) but not in Experiment 1, where the dogs heard both full speech and vocoded speech. These results can be explained by the amount of context available to the dogs in each study. In Experiment 1, dogs hear four blocks of stimuli consisting of the full-speech and vocoded versions of their name and a foil; as such, they hear the full speech version of their name and the foil name at least by the end of the first block. As such, dogs could use the information from the full speech version of their name to better understand the vocoded version. Improved comprehension of vocoded speech after presentation of the clear version of the speech is known as *pop-out*, and this phenomenon has been demonstrated in adult humans (Davis et al., 2005). The full-speech information can then be used to facilitate perception throughout the remainder of the trials. Given that all dog participants had the full speech cues available to them, the number of phonemes in their name was less important context than the full speech information. Similarly, in Heimbauer et al. (2011), an analysis of Panzee’s performance demonstrated no effect of syllable number in a similar paradigm where Panzee heard both natural speech and noise-vocoded speech. However, in Experiment 2, the full speech cues were no longer available, since all the stimuli were vocoded. As such, dogs had to solely rely on cues available in the vocoded speech to differentiate their name from the foil names. Dogs with more phonemes in their name had more phonemic information available to them in the vocoded speech. Studies in adult humans show that the more context available for the listener in noise-vocoded speech, the easier it is to understand the speech (Dahan and Mead 2010; Sheldon et al. 2008). Since dogs with more phonemes in their name are provided with more acoustic context, the increased context could allow for easier comprehension of their name and better differentiation of their name and the foils.

One significant difference between this dog study and prior studies with primates is the number of channels used in the vocoded speech. The more channels in the vocoded speech, the closer it is to natural speech, and the easier it is to comprehend for both adults (Friesen et al. 2001) and children (Newman et al. 2015). The 16-channel noise-vocoded speech used in this study was a level at which chinchillas failed to recognize trained words (Shofner et al. 2018). With prior exposure to vocoded speech, Kanzi the bonobo was able to recognize familiar words in a more difficult 7-channel vocoded speech (Lahiff et al. 2022), and Panzee the chimpanzee has achieved above-chance recognition of familiar words in as low as 3-channel vocoded speech (Heimbauer et al. 2021). Additionally, in her initial study, Panzee was able to recognize 7-channel vocoded speech with no prior exposure (Heimbauer et al. 2011). Without prior exposure to noise-vocoded speech, toddlers recognize familiar words in vocoded speech down to 8 channels (Newman et al. 2015). As this study only used sixteen-channel vocoded speech, it is unknown whether dogs possess the ability to recognize equivalently degraded speech. Future studies can examine the lowest number of channels of familiar noise-vocoded speech that dogs can recognize, and compare this to the infant results. Dogs can also be exposed to noise-vocoded speech and then tested on word recognition, as was done in the bonobo study and second chimpanzee study, for better comparison with the language-trained apes. These studies could provide information about whether recognition of degraded speech came about via convergent evolution, in which the trait arose independently in these multiple evolutionary branches for mechanistic or functional purposes, or via homologous evolution, in which the shared trait arose in an ancestor of dogs and primates (Fitch 2017).

Vocoded speech differs from typical speech primarily in terms of its spectral properties: as opposed to the fine spectral detail of natural speech, vocoded signals can be considered a “rough approximation” spectrally. Speech can be degraded in other ways as well. For example, to create sine-wave analogs to speech, the first three or four resonant energy bands in the original signal are each replaced with a time-varying sinusoid (Remez et al. 1981). This maintains the global dynamic spectral structure of the peaks of the power spectrum, but removes information in the spectral valleys, or, to put it another way, it results in a signal that lacks the resonant properties of the human vocal tract but maintains the time-varying spectral properties. Some researchers have discussed sine-wave analogs and noise-vocoded speech as being opposites: one blurs the spectral details, and the other sharpens those details. Humans are successful at recognizing both types of signals quite well, suggesting that there may not be any one set of cues that must be present for recognition. Future research could explore how well dogs recognize such other forms of degradation as well.

## Conclusion

This set of studies demonstrated that dogs can recognize a 16-channel vocoded version of their name without any prior exposure to vocoded speech, and without immediate exposure to the natural speech version of the vocoded name. However, recognition was dependent upon the number of phonemes in the dogs’ names, suggesting that the amount of information present in the name when vocoded played a role in dogs’ ability to recognize their name. Future studies could explore other forms of degradation or could determine the lowest number of channels at which dogs can recognize their name, which would allow for better comparisons with other non-human animal studies on this topic. These studies are a step towards understanding the evolutionary development of human speech perception.

## Electronic supplementary material

Below is the link to the electronic supplementary material.

Supplementary Material 1

Supplementary Material 2

Supplementary Material 3

## Data Availability

Please email the authors for access to data and materials.
