# Supplementary material for: Perception of vocoded speech in domestic dogs
Source: Anim Cogn. 2024 Apr 16;27(1):34. doi: 10.1007/s10071-024-01869-3 (PMC11021312; doi:10.1007/s10071-024-01869-3)
Supplement: Supplementary file 1 — Supplementary Material 1 [file 10071_2024_1869_MOESM1_ESM.docx]

Supplementary Audio

The first clip is a natural speech recording of the dog name “Nella”.

The second clip is a 16-channel vocoded version of the dog name “Nella”.

Supplementary Tables

Supplementary Table 1: Dog demographics for Experiment 1

| Dog | Drop? | Sex | Age | Breed Description | Length of Time With Name | Drop Reason |
| --- | --- | --- | --- | --- | --- | --- |
| Beaux | yes | M | 3 | Whippet mix | 2 | Owner interference |
| Beiya | yes | F | 11 | Beagle/Shepherd | 11 | Noise outside testing room |
| Chief | yes | M | 2 | Pit Bull mix | 1.5 | People talking outside testing room |
| Bruno | no | M | 8 | Golden Retriever | 8 |  |
| Cap | no | M | 3.5 | Golden Retriever | since 8 weeks old |  |
| Clementine (Clem) | no | F | 12 | Aussie mix \ Unknown | since 3 months old |  |
| Clouseau (Clou) | no | M | 2.5 | Frenchie | 2.25 |  |
| Ethel | no | F | 2 | Standard Poodle | 11 months |  |
| Harper | no | M | 4 | Mountain Cur Mix | 4 |  |
| Hector | no | M | 8 | West Highland White Terrier | since 9 weeks |  |
| Humphrey | no | M | 1.3 | Beagle/Catahoula Leopard Dog | 1 year 3 months |  |
| Juno | no | F | 5 | Labrador Retriever | 5 |  |
| Lexi | no | F | 2.9 | Pitbull mix | since 6 weeks |  |
| Lilly | no | F | 9 | Shih Tzu | 9 |  |
| Lily | no | F | 1.5 | Spaniel mix | 1.5 |  |
| Maddie | no | F | 1 | Aussie/Papillon mix | 1 |  |
| Nella | no | F | 4.5 | Lab / Pitbull mix | 3 |  |
| Piper | no | F | 2.5 | Mix | 2.5 |  |
| Prince | no | M | 5.5 | Golden Retriever | 5.5 |  |
| Rocket | no | M | 2.5 | Border Collie / Retriever mix | 2 |  |
| Ruby | no | F | 1.5 | German Shorthaired Pointer | Since 8 weeks |  |
| Splash | no | M | 7 | Labrador Mix | 6+ |  |
| Stella | no | F | 5 | Golden Retriever | 5 |  |
| Stella (#2) | no | F | 1.2 | Corgi and Blue Heeler mix | 1 year 2 months |  |
| Taters | no | M | 10 | Pomeranian mix | 3.5 |  |
| UV (Violet) | no | F | 4 | Mix | 3.5 |  |
| Wentworth | no | M | 2.3 | Coton de Tulear | 1.5 |  |
| Ziggy | no | F | 2.5 | Irish setter doodle | 2.5 |  |

Supplementary Table 2: Dog demographics for Experiment 2

| Dog | Keep | Sex | Age | Breed Description | Length of Time With Name | Drop Reason |
| --- | --- | --- | --- | --- | --- | --- |
| Ollie | no | M | 0.75 | Parson Russell Terrier | 7 months | Dog not old enough |
| Bailey | no | F | 5 | Mix | 5 | Dog not willing to participate |
| Dug | no | M | 4 | Mix | 3.5 | Dog not willing to participate |
| Hazel | no | F | 1 | Chihuahua Mix | 0.75 | Dog didn’t have name long enough |
| Koa | no | F | 3 | Labrador Mix | 0.75 | Speaker malfunction and dog didn’t have name long enough |
| Piper | no | F | 1.5 | Toy Poodle | 1.5 | Speaker malfunction |
| Abbey | yes | F | 7 | Labrador | 7 |  |
| Bentley | yes | M | 8 | Brussels Griffon Mix | 8 |  |
| Charlie | yes | F | 2 | Beagle | 1.7 |  |
| Cooper | yes | M | 2.58 | Cockapoo | 2.25 |  |
| Corbett | yes | M | 7.5 | Boxer mix | 7 |  |
| Corby | yes | F | 4 | Irish Wolfhound | 4 |  |
| Cricket | yes | F | 2 | Chihuahua | 2 |  |
| Ernie | yes | M | 1.92 | Boston Terrier | 1.5 |  |
| Gracie | yes | F | 1 | Labrador | 15 months |  |
| Harper | yes | F | 1.33 | Collie / Labrador mix | 1.08 |  |
| Izzy | yes | F | 2 | Labrador / Poodle mix | 2 |  |
| Jack | yes | M | 4 | Staffordshire Terrier mix | 4 |  |
| Jackson | yes | M | 10 | Beagle/Coonhound mix | 9.5 |  |
| Jasper | yes | M | 9 | Pug / Beagle / Pit Bull mix | 5 |  |
| Kirby | yes | M | 1 | Labrador | 1 |  |
| Luna | yes | F | 7 | Beagle | 5 |  |
| Mabel | yes | F | 1.5 | Poodle mix | 17 months |  |
| Red | yes | M | 4 | Greyhound | 6 |  |
| Roosevelt (Rosie) | yes | F | 1 | Shih Tzu / Toy Poodle mix | 1 |  |
| Shelby | yes | F | 1 | Bernese Mountain Dog / Poodle mix | 11 months |  |
| Smitty | yes | M | 1.5 | Mix | 1.25 |  |
| Tag | yes | M | 4 | Pembroke Welsh Corgi | 4 |  |
| Tiger | yes | M | 3 | Pit Bull mix | 2.5 |  |
| Tilghman | yes | M | 6 | Bichon / Poodle mix | 5 |  |
| Toby | yes | M | 4 | Yorkshire Terrier mix | 4 |  |
